# Mathematical models of the colonic microbiota: an evaluation of accuracy using *in vitro* fecal fermentation data

**DOI:** 10.3389/fnut.2025.1623418

**Published:** 2025-09-25

**Authors:** Vitor Geniselli da Silva, Nick William Smith, Jane Adair Mullaney, Nicole Clémence Roy, Clare Wall, Warren Charles McNabb

**Affiliations:** ^1^Riddet Institute, Massey University, Palmerston North, New Zealand; ^2^High-Value Nutrition National Science Challenge, Auckland, New Zealand; ^3^AgResearch, Palmerston North, New Zealand; ^4^Department of Human Nutrition, University of Otago, Dunedin, New Zealand; ^5^Department of Nutrition and Dietetics, The University of Auckland, Auckland, New Zealand

**Keywords:** gut microbiota, modeling, *in silico*, correlation, short-chain fatty acid

## Abstract

Traditional approaches for studying diet-colonic microbiota interactions are time-consuming, resource-intensive, and often hindered by technical and ethical concerns. Metagenome-scale community metabolic models show promise as complementary tools to overcome these limitations. However, their experimental validation is challenging, and their accuracy in predicting colonic microbial function under realistic dietary conditions remains unclear. This study assessed the accuracy of the Microbial Community model (MICOM) in predicting major short-chain fatty acid (SCFA) production by the colonic microbiota of weaning infants, using fecal samples as a proxy. Model predictions were compared with experimental SCFA production using *in vitro* fecal fermentation data at the genus level. The model exhibited overall poor accuracy, with only a weak, significant correlation between measured and predicted acetate production (*r* = 0.17, *p* = 0.03). However, agreement between predicted and measured SCFA production improved for samples primarily composed of plant-based foods: acetate exhibited a moderate positive correlation (*r* = 0.31, *p* = 0.005), and butyrate a trend toward a weak positive correlation (*r* = 0.21, *p* = 0.06). These findings suggest that the model is better suited for predicting the influence of complex carbohydrates on the colonic microbiota than for other dietary compounds. Our study demonstrates that, given current limitations, modeling approaches for diet-colonic microbiota interactions should complement rather than replace traditional experimental methods. Further refinement of computational models for microbial communities is essential to advance research on dietary compound-colonic microbiota interactions in weaning infants.

## 1 Introduction

The relationship between dietary compounds and colonic microbiota has garnered scientific interest due to its impact on host health (1, 2). From a health perspective, changes in colonic microbial function are more relevant than alterations in composition. Imbalances in microbial metabolite production may distinguish individuals with disease from healthy controls, despite individual variations in microbial taxonomy (3–6). Numerous metabolites play crucial roles in the bidirectional communication between the colonic microbiota and the host, such as neurotransmitters, polyamines, vitamins, bile acids, and organic acids (7). Among them, the short-chain fatty acids (SCFAs), acetate, propionate, and butyrate, offer numerous benefits to the host, including maintaining colonic barrier integrity, serving as an energy source for colonocytes, and exerting neuroprotective effects (8–11).

Most of our current understanding about diet-colonic microbiota interactions centers on the effects of complex carbohydrates on the adult microbiota. In contrast, the impact of other dietary compounds, such as fatty acids and polyphenols, remains underexplored, particularly in underrepresented populations such as infants and older adults (12). A better understanding of how dietary compounds affect colonic microbial function across diverse human populations is crucial. Clinical trials are standard approaches for evaluating this impact and assessing potential health outcomes from diet-microbiota interactions. However, they are time- and resource-consuming, and often challenged by technical and ethical concerns (13). Given these limitations, mathematical models show promise as complementary tools to reduce the cost and time of microbiota investigations (14, 15).

Various models have been proposed to investigate diet-colonic microbiota interactions, including kinetic-based (16–19), agent-based (20–22), and genome-scale metabolic models (GEMs) (23–26). GEMs use metabolic reconstructions, a mathematical representation of a microorganism’s metabolism, and flux balance analysis (FBA) to predict microbial metabolite production as fluxes (units of concentration per time) (27). Metagenome-scale community metabolic models (MGCMs) extend this concept to microbial communities (14, 15, 28). Among MGCMs, the Microbial Community model (MICOM) stands out for its user-friendly approach, extensive documentation, and pre-made workflows that range from data preparation to visualization (14, 29).

However, experimentally validating the predictions of MGCMs is challenging (30). A recent study compared measured SCFA fluxes from *ex vivo* fecal incubations with predicted fluxes obtained from MICOM under the influence of dietary fibers (31). An agreement between predictions and experimental measurements was reported for propionate and butyrate. Nevertheless, while this study partially validated *in silico* predictions for the influence of isolated dietary fibers, the model’s accuracy in predicting how whole foods shape colonic microbial function remains unexplored. Importantly, foods contain multiple dietary compounds that can interact during digestion and modulate their collective impact on colonic microbes (32, 33).

This study aimed to evaluate MICOM’s accuracy in predicting microbial SCFA production in real-life feeding scenarios for weaning infants. Predicted acetate, propionate, and butyrate fluxes were compared with fluxes measured experimentally from a published *in vitro* fecal fermentation study (34). The *in silico* simulations were designed to match the experimental setup as closely as possible, which examined how complementary foods, alone or combined with infant formula and/or other foods, affected major SCFA production by the colonic microbiota of weaning infants.

## 2 Methods

### 2.1 Experimentally measured fluxes of short-chain fatty acids

Measured acetate, propionate, and butyrate fluxes were estimated using data from a fecal fermentation study (34). The study evaluated the effects of complementary foods on the composition and SCFA production of the colonic microbiota in weaning infants, using fecal samples as a proxy. Food ingredients were tested individually and in combination with other foods, infant formula, or both, resulting in 53 samples. Samples were digested *in vitro* using a static model adapted from the INFOGEST protocol (35) to mimic the gastrointestinal conditions of 6-month-old infants, then fermented for 24 h at 37 °C using a pooled fecal inoculum from six healthy weaning infants (aged 5–11 months).

Organic acids were acidified with hydrochloric acid, extracted with diethyl ether, derivatized with N-tert-butyldimethylsilyl-N-methyltrifluoroacetamide, and detected using gas chromatography with a flame ionization detector [see methods description in (34)]. A solution of 2-ethyl butyric acid was used as an internal standard to account for the batch variations. Organic acids were quantified using standard solutions of acetate, propionate, and butyrate. The production of SCFAs was determined for each sample as the difference between measured SCFAs before and after fermentation, normalized by the dry weight of the fermented sample. Fluxes of acetate, propionate, and butyrate were calculated by dividing the concentration of each organic acid by the fermentation time (24 h).

### 2.2 Software

Simulations were conducted in Python (version 3.9.10) within the Spyder integrated development environment (version 5.4.2) using MICOM (14) (version 0.37.0) and the CPLEX Optimization Studio solver (IBM ILOG, version 22.1), which was accessed under an academic license. Additionally, the Assembly of Gut Organisms through Reconstruction and Analysis version 2 (AGORA) (36) metabolic reconstructions were employed to infer the metabolism of the infant fecal microbiota. Data and code are available at https://github.com/vgenisel/Assessing-the-accuracy-of-a-gut-microbiota-modelling-tool.

### 2.3 *In silico* media design

Media for the simulations were designed following the workflow of a previous study that used MICOM to assess the impact of complementary foods on the fecal microbiota of weaning infants (37). In short, the “Design a diet” function of the Virtual Metabolic Human database was used to select the foods composing the media (38). Foods were chosen to match the experimental samples as closely as possible (Supplementary Table 1). Additionally, the *in silico* media were designed to have the same dry mass of foods (150 g) to replicate the experimental conditions, which used 1.5 g of freeze-dried foods (a scalar increase was necessary to mitigate numerical instability in the simulations). The same approach was used to design media composed of food combinations, including foods with other foods, infant formula, or both, keeping the same ratios used in *in vitro*: 50% food1 with 50% food2; 20% food with 80% infant formula; and 10% food1 with 10% food2 and 80% infant formula.

Imported data were processed through the MICOM workflow for media design (31, 37), which added host-secreted compounds (mucin cores and bile acids), removed diluted compounds that are absorbed in the small intestine, and supplemented the media with minimal missing nutrients to ensure a community growth rate of 0.3/h. Finally, media compounds were diluted by a factor of 10 to match experimental conditions, where approximately 10% of the volume of post-dialysis digested food samples was fermented with fecal inoculum (34). As MICOM’s workflow does not directly account for digestion, the composition of the *in silico* media was assumed to reflect the chemical profile of the experimentally digested food samples.

### 2.4 *In silico* simulations

The relative abundance of the microbial community used in the simulations reflects baseline values from the *in vitro* fecal fermentation study (34) (Supplementary Table 2). Simulations were performed at the genus level due to the use of 16S rRNA gene sequencing in the experimental work. Briefly, raw paired-end sequencing data were generated by amplifying the V3-V4 regions of the 16S rRNA gene using an Illumina MiSeq platform. Primers were removed using Cutadapt (version 2.3) (39) and Trimmomatic (40). The DADA2 (version 1.32) (41) pipeline was followed for denoising, read truncation, chimera removal, and inferring amplicon sequence variants. The SILVA database (version 138.1) (42) was used for taxonomy assignment, and amplicon sequence variants were collapsed at the genus level using the microbiome (version 1.26) (43) package. Only genera with at least 0.001 relative abundance were included in the simulations to reduce numerical instability and processing time. A total of 31 genera (out of 54) were included, representing 99.3% of the relative abundance of the microbial community. Pan models of the AGORA2 metabolic reconstructions (36) for these genera were built by pooling microbial metabolic strains into higher taxonomic ranks.

Simulations followed published protocols (31, 37). MICOM is based on FBA under a mass steady-state assumption (14), representing the exponential phase of microbial growth, during which growth rates remain constant. Fluxes of microbial metabolites are calculated as the solution to a constrained linear programming problem, integrating the biochemical reactions performed by the microbial community, assuming no accumulation of substrates in the system, to maximize microbial community biomass. Notably, MICOM incorporates a trade-off between maximal community growth and maximal individual microbial growth (14). This strategy prevents the most abundant microbes from growing at the expense of low-abundance ones. The optimal cooperative trade-off was determined for each *in silico* medium (values ranged from 0.4 to 0.7) using MICOM’s “tradeoff” function. Additionally, MICOM employs a linearization strategy that relates the growth rates of individual taxa to their relative abundance (14). Consequently, the microbial community was expected to exhibit consistent growth patterns across different *in silico* media, with high-abundance genera predicted to have higher growth rates than those of lower abundance. Importantly, microbial relative abundance was used as a proxy for microbial biomass in the simulations, and fluxes of microbial metabolites were normalized by the dry weight of microbial biomass (expressed in millimoles per gram per hour {mmol/gDW h}).

### 2.5 Statistical analyses

To account for dissimilarities between the design of *in silico* and *in vitro* studies, standard scores (z-scores) were calculated for measured and predicted fluxes (Supplementary Table 3). The z-score describes the number of standard deviations a value differs from the mean. This strategy enables a comparison of results across studies with different designs (31). Pearson correlation coefficients (r) and two-tailed *p*-values (p) between measured and predicted z-scores for acetate, propionate, and butyrate production were calculated in Python (version 3.10.9) using pandas (version 2.2.3) (44) and SciPy (version 1.10.0) (45). Plotnine (version 0.14.5) was used to plot the correlations (46). To further assess the agreement between predicted and measured z-scores, the Bland–Altman analysis (47) was performed, and the 95% limits of agreement (mean difference ± 1.96 standard deviations) were calculated using the packages NumPy (version 1.23.5) (48) and matplotlib (version 3.10.0) (49). The normal distribution of the data was verified through the Shapiro–Wilk test using SciPy (version 1.10.0) (45). Heatmaps and radar charts were generated using matplotlib (version 3.10.0) (49), seaborn (version 0.13.2) (50), and SciPy (version 1.10.0) (45).

## 3 Results

### 3.1 Pearson correlations between predicted and measured SCFA production

Correlation analyses demonstrated a weak agreement between predicted and measured acetate production (*r* = 0.17, *p* = 0.03). However, this was the only significant correlation (*p* < 0.05) observed when considering the entire dataset (Supplementary Figure 1). To investigate whether combining food ingredients with other dietary compounds would impact the accuracy of the model, subsequent analyses clustered samples into the following categories: food ingredients alone, foods combined with infant formula (food-formula combinations), foods combined with other foods (food-food combinations), and foods combined with both (food-food-formula combinations). A trend toward a weak correlation was observed between predicted and measured butyrate production for food ingredients alone (*r* = 0.28, *p* = 0.07). Additionally, a moderate correlation was observed between model predictions and experimental productions of acetate and total SCFAs for food-food combinations (*r* = 0.43 and 0.41, with *p* = 0.006 and 0.01, respectively), while propionate exhibited a trend toward a weak negative correlation (*r* = −0.30, *p* = 0.06; Supplementary Figure 2). In contrast, no significant correlations were observed between predicted and measured z-scores for food-formula and food-food-formula combinations (Supplementary Figure 2).

**Figure 1:**
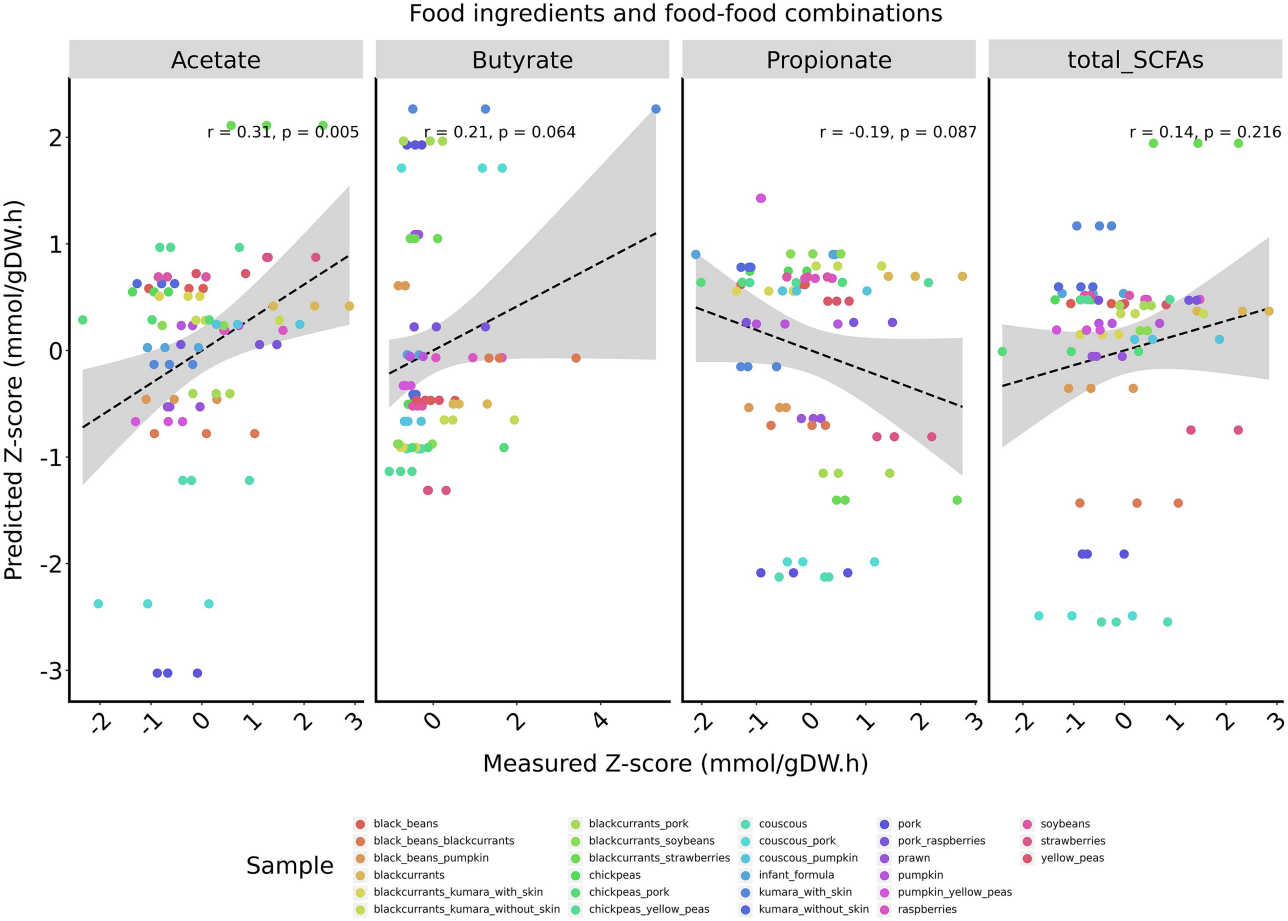
Pearson correlations between measured and predicted z-scores of major SCFAs for food ingredients and food-food combinations. SCFAs are displayed from left to right: acetate, butyrate, and propionate. Total SCFAs correspond to the sum of acetate, propionate, and butyrate. Pearson correlation coefficients (r) and two-tailed *p*-values are calculated for each plot individually. A regression line is shown in black, with the corresponding 95% confidence interval in gray.

Given that combining infant formula with foods reduced the model’s accuracy, an analysis was performed using only food ingredients and food-food combinations, corresponding to samples predominantly composed of plant-based foods. For this subset of samples, acetate exhibited a moderate agreement between predicted and measured z-scores (*r* = 0.31, *p* = 0.005), butyrate demonstrated a trend toward a weak positive correlation (*r* = 0.21, *p* = 0.06), and propionate showed a trend toward weak a negative correlation (*r* = −0.19, *p* = 0.08; Figure 1). Similar results were observed when animal-based food samples were completely excluded from the dataset (Supplementary Figure 3).

### 3.2 Bland–Altman analysis

**Figure 2:**
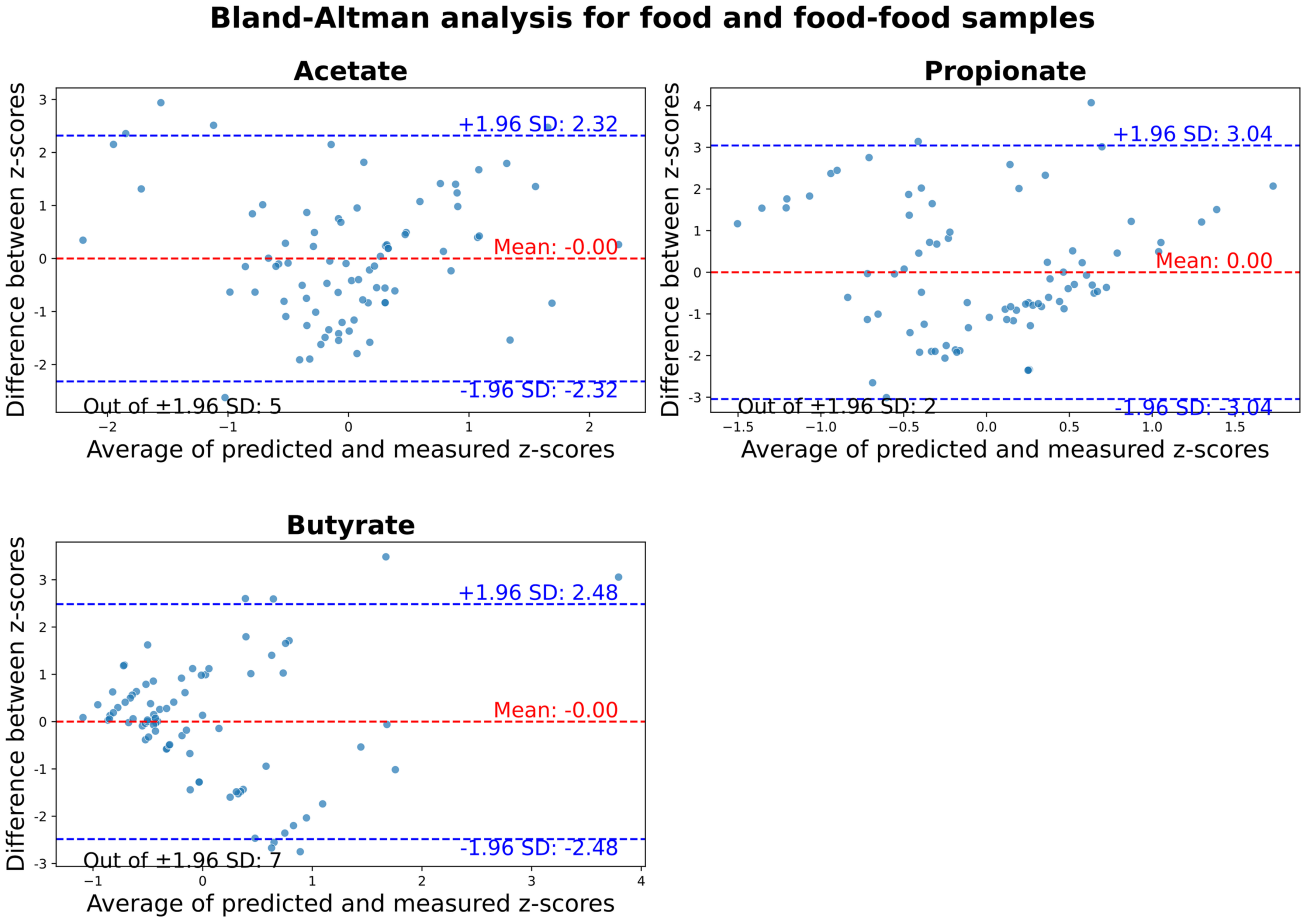
Bland–Altman plots comparing predicted and measured z-scores of major SCFAs for food ingredients and food-food combinations. SCFAs are displayed from left to right: acetate, propionate, and butyrate. The red line represents the mean difference between z-scores, while the blue lines indicate the upper and lower 95% limits of agreement (mean difference ± 1.96 standard deviation). Each sample is depicted as a green dot.

The normality of the dataset, comprising food ingredients and food-food combinations, was verified by the Shapiro–Wilk test. While the differences between predicted and measured z-scores for individual SCFAs followed a normal distribution (*p*-value > 0.05), their sum did not (*p* = 0.01; Supplementary Table 4). Consequently, Bland–Altman plots were generated only for acetate, propionate, and butyrate individually (Figure 2). Since fluxes were standardized into z-scores, all plots’ mean differences between z-scores were zero. Among the SCFAs, acetate exhibited the lowest limits of agreement, indicating better concordance between predicted and measured z-scores. Overall, most samples fell within the 95% limits of agreement for the major SCFAs. However, some samples exceeded these limits: 5 out of 81 for acetate, 2 out of 81 for propionate, and 7 out of 81 for butyrate. This suggests that the model had limitations in accurately predicting experimental outcomes across the entire dataset.

### 3.3 Comparison between predicted and measured z-scores

**Figure 3:**
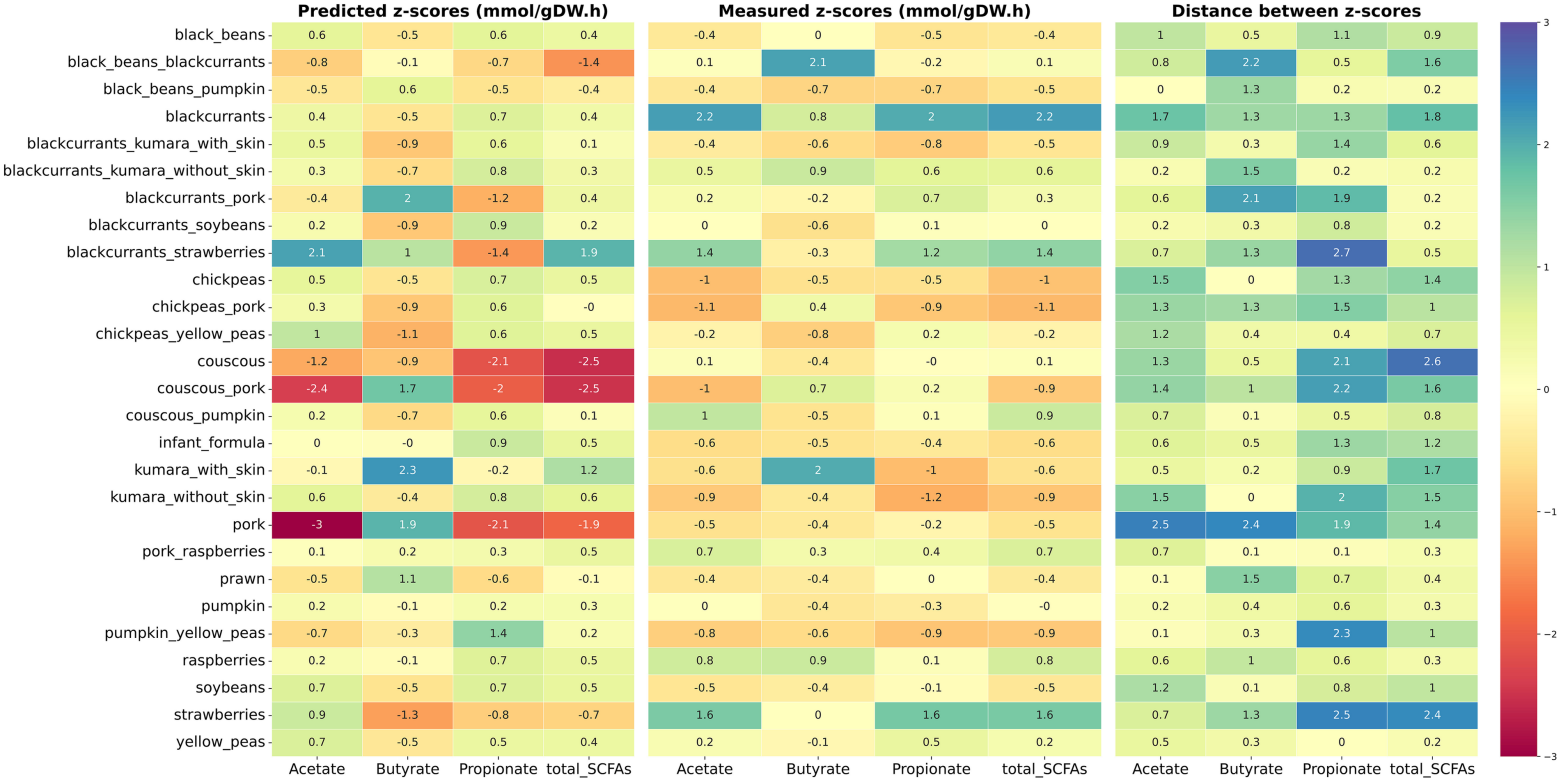
Heatmap of measured and predicted z-scores of major SCFAs for food ingredients and food-food combinations. Predicted z-scores obtained via *in silico* modeling are displayed on the left and measured z-scores obtained via *in vitro* fermentation on the middle. The absolute difference between predicted and measured z-scores is displayed on the right (distance between z-scores). Cells are colored by intensity, with the lowest values in red and the highest values in blue.

To better visualize the comparison between *in silico* with *in vitro* outcomes, a heatmap (Figure 3) and a radar chart (Supplementary Figure 4) were plotted. Only food ingredients and food-food combinations were included, as they demonstrated better agreement between predicted and measured values. Results showed several disagreements between *in silico* predictions and experimentally measured z-scores for these samples. For instance, black beans combined with blackcurrants had the greatest experimental production of butyrate among food-food combinations. In contrast, the model predicted the greatest butyrate production for pork combined with blackcurrants or couscous. For food ingredients, notable discrepancies were observed in the z-scores for chickpeas, couscous, soybeans, and kūmara samples, with only the butyrate prediction matching the experimental outcomes. Furthermore, the model did not accurately predict the ability of strawberries to increase propionate and total SCFA production *in vitro*. On the other hand, the model satisfactorily predicted the greatest production of butyrate for kūmara with skin among the food ingredients and the greatest production of acetate and total SCFAs for blackcurrants combined with strawberries among the food-food combinations. Additionally, the model captured the impact of blackcurrants and raspberries in increasing the total SCFA production compared to other foods.

### 3.4 Sensitivity analyses

To assess whether prediction quality depends on input microbial composition, additional simulations were conducted using post-fermentation relative abundances for each sample. Consistent with our initial results, the model exhibited poor overall agreement with experimental outcomes, with no significant correlations observed between predicted and measured z-scores for all samples. However, acetate production showed a moderate positive correlation (*r* = 0.42, *p* = 0.008) and total SCFA production trended positively (*r* = 0.28, *p* = 0.08) when analyses were restricted to food-formula combinations (Supplementary Figure 5). Similar to the initial results, food-formula combinations involving protein-rich foods, such as pork-formula and prawn-formula, showed the greatest discrepancies between predicted and measured outcomes (Supplementary Figures 6, 7).

## 4 Discussion

This study compared the predicted production of acetate, propionate, and butyrate *in silico* using MICOM (14) with experimental values obtained from a fecal fermentation study (34). Our focus on assessing the model’s accuracy in predicting the production of health-relevant metabolites, rather than microbial growth, was driven by the lack of available data on *in vitro* individual microbial growth rates. Additionally, from a health perspective, microbial function is more informative than composition, as colonic microbes are functionally redundant, and the colonic microbiota of healthy individuals maintains similar functionality despite taxonomic differences (3, 6). The workflows for *in silico* simulations and statistical comparison between *in silico* and *in vitro* outcomes followed published protocols (31, 37). Another strength was the assessment of the model’s accuracy in predicting SCFA production by the colonic microbiota of weaning infants under realistic infant feeding patterns, including whole foods, foods combined with other foods, infant formula, or both.

When evaluating all samples, the model demonstrated poor accuracy, with only acetate showing a weak correlation between predicted and measured outcomes. Interestingly, we observed increased accuracy when analyzing samples predominantly composed of plant-based foods. Acetate exhibited a moderate positive correlation, while propionate and butyrate showed a trend toward weak positive and weak negative correlations, respectively. These findings suggest that MICOM’s accuracy in predicting the function of the infant microbiota increases for media enriched in complex carbohydrates. This result is expected, considering that the influence of complex carbohydrates on colonic microbes is far better understood than that of other dietary compounds. Consequently, metabolic reconstructions of colonic microbes are often not curated for biochemical reactions involving amino acid and lipid utilization due to limited available data (51). Additionally, this result is likely driven by the functional capacity of the colonic microbiota of weaning infants. Observational studies have demonstrated that the weaning infant microbiota transitions from primarily degrading human milk oligosaccharides to metabolizing complex carbohydrates, while amino acid fermentation increases but remains less prominent compared to that of the adult colonic microbiota (52, 53).

Given that correlation analyses assess the strength of the relationship between two variables but do not indicate how these variables differ from each other, a Bland–Altman analysis was conducted to determine the limits of agreement (95% confidence interval) between predicted and measured SCFA production. Standard scores were used to account for differences in magnitude and unit between methods (54). Acetate had lower limits of agreement than propionate and butyrate, suggesting stronger concordance between predicted and measured z-scores, which aligns with the correlation results. Overall, most food and food-food combination samples fell within the 95% limits of agreement. However, some exceptions highlighted the model’s limited ability to accurately predict SCFA experimental production across the entire dataset.

Among food ingredients and food-food combinations, disagreements were observed between *in vitro* and *in silico* outcomes. For example, strawberries and the combination black beans-blackcurrants had positive z-scores for total SCFA production *in vitro* but negative z-scores *in silico*. On the other hand, some samples that drove the greatest total SCFA production *in vitro*, such as blackcurrants, raspberries, and the blackcurrants-strawberries combination, also resulted in high positive z-scores *in silico*. Sensitivity analyses assessing the impact of microbial composition on prediction accuracy indicated that community composition strongly influences simulation outcomes. However, the model consistently showed limited predictive performance across SCFAs and samples, with acetate showing better agreement in carbohydrate-rich samples. As a take-home message, our study demonstrates that emerging modeling approaches for diet-colonic microbiota interactions are imperfect and should not replace experimental methods. Instead, given their cost- and time-efficiency, and ability to leverage existing data, these models offer a valuable starting point for generating insights that can guide the design of *in vitro* and *in vivo* studies. Their further refinement and use as complementary tools represent a promising opportunity to advance diet-colonic microbiota research.

Future directions for the development of MGCMs include expanding the number of high-quality microbial metabolic reconstructions, which should be built using sequencing data that meet quality standards and subsequently curated with experimental data (55–57). Shotgun metagenomic sequencing data is preferable to 16S rRNA sequencing data, as it more accurately captures the metabolic potential of colonic microbes (58). Importantly, experimental data remain crucial for model curation and validation (59), with a notable need for more research into the behavior of colonic microorganisms in response to various dietary compounds. The accuracy of MGCMs could also benefit from incorporating omics data, such as metatranscriptomics, to better personalize the model’s conditions (60). Additionally, integrating dynamic FBA could improve the representation of changes in the colonic environment over time (24, 61). Finally, databases used for designing media in *in silico* simulations require further refinement to better account for the heterogeneity of dietary patterns across individuals.

Our results partially contrast with a previous study that reported agreement between MICOM’s prediction for propionate and butyrate and experimental outcomes from *ex vivo* fecal incubations with isolated dietary fibers using adult inoculum (31). The modest performance observed here is likely due to the different study designs. The fecal microbiota of weaning infants has distinct functionality compared to the adult microbiota, including a higher proportional production of acetate and lower of propionate and butyrate (62, 63). Furthermore, whole foods are complex matrices containing not only fiber but also other compounds, such as protein, fat, and phytochemicals, all of which impact the function of colonic microbes (64–66). Finally, while the referenced study used metagenomic data to build models at the species level and normalized SCFA predictions based on microbial biomass (31), our simulations were limited to the genus rank and did not incorporate biomass normalization due to using 16S rRNA sequencing data, thereby reducing metabolic specificity. This contrasting result highlights the need for further investigations of the model predictive accuracy across different study conditions and host populations, particularly concerning the production of health-relevant metabolites like butyrate (8, 9).

A major limitation of our study is the difference in outcomes obtained from distinct methods, as fluxes predicted *in silico* are not equivalent to the concentration of metabolites measured *in vitro*. To address this limitation, we followed a published protocol that used standard scores to compare results from different methodologies and validate MICOM’s predictions of propionate and butyrate production (31). However, quantifying SCFA fluxes using static *in vitro* fermentation is a limited representation, as metabolites accumulate and substrates are depleted over time (67). Over long fermentation periods, these conditions diverge from the dynamic conditions and steady state assumption inherent in FBA-based models, which assumes no accumulation of substrates in the system (27).

Additionally, the accuracy of models based on FBA strongly depends on the quality of the metabolic reconstructions used in the simulations. A recent systematic evaluation of FBA-based tools, including MICOM, reported low prediction accuracy of microbial growth rates compared to experimental data when using the AGORA metabolic reconstructions (68). The authors highlighted that semi-curated metabolic reconstructions were not sufficiently accurate for predicting the behavior of microorganisms (68). Our simulations were performed using the second version of AGORA, which was generated via a semi-automated pipeline and manually refined (36). However, it is important to recognize that these reconstructions may contain gaps or inaccurately assigned biochemical reactions (51).

Another limitation was the use of pooled fecal data, justified by the absence of individual-level data in the *in vitro* fermentation study. Although pooling fecal samples is a common practice in such studies, it inevitably alters the original microbial community structure, reducing inter-individual variability and functional resolution. These changes are likely to influence microbial functional dynamics in ways that are not captured by the taxonomic profile of the pooled sample (69, 70). Using amplicon 16S sequencing data limited model construction to the genus level, potentially masking metabolic differences between microbial species (31, 71). Additionally, this approach reduced the number of taxa included in the simulations and hindered the normalization of SCFA predictions by bacterial biomass. Notably, bacterial species that are present in low abundance yet biologically meaningful (keystone species) may be underrepresented when using 16S rRNA sequencing data (72, 73). Moreover, relying on microbial relative abundances as a proxy for absolute quantities, which can be biased by interdependencies between taxa and may not accurately reflect the true composition of the microbial community (74), can influence the model predictions. To overcome this limitation, simulations should be based on absolute microbial biomass, estimated via metagenomics or sequencing combined with qPCR (75, 76).

Furthermore, the *in silico* media were designed using the Virtual Metabolic Human database (38), which lacks information on diverse cooking methods and food ingredients. Notably, indigenous foods available in New Zealand and used in the experimental work (34), such as kūmara (sweet potato variety), were not included in the database and had to be substituted with the most similar available food. Finally, while the *in vitro* study used a static protocol to mimic the digestion and absorption of dietary compounds (34), MICOM, like other diet-microbiota models, currently does not account for digestion. Instead, it represents absorption by diluting the dietary compounds identified to be absorbed by the human intestine using a scalar (14). Not accounting for digestion is a potential major source of variation between *in silico* and *in vitro* outcomes, as digestion influences food structure and, consequently, the nutrient profile accessible for microbial fermentation (77). However, this limitation is intrinsic to the modeling framework, as computationally simulating food digestion is challenging. Although promising tools are emerging (78), they have not yet been integrated into MGCMs.

## 5 Conclusion

This study evaluated the accuracy of the metagenome-scale community metabolic model MICOM in predicting SCFA production by the fecal microbiota of weaning infants under realistic complementary infant feeding patterns, using static *in vitro* fecal fermentation data as a comparator. A weak positive correlation was observed between predicted and measured acetate production. The agreement between predicted and measured SCFA fluxes improved when analyzing samples predominantly composed of plant-based foods. These findings suggest that the model more accurately replicates experimental results when simulating media rich in complex carbohydrates. Despite disagreements between experimental and simulated outcomes for specific SCFAs, the model identified samples with the highest total SCFA production *in vitro*. This exemplifies the model’s limitations as a replacement for traditional experimental methods but supports its potential as a complementary tool. Further model development is essential to improve its accuracy, particularly for media-rich in fat and protein. Refined versions of the model would contribute to advancing research on the relationship between diet and microbiota.

## Data Availability

The original contributions presented in the study are publicly available. This data can be found here: https://www.ncbi.nlm.nih.gov/bioproject/PRJNA1327581 [SRA database BioProject Accession number PRJNA1327581].
