# Supplementary material for: Mathematical models of the colonic microbiota: an evaluation of accuracy using in vitro fecal fermentation data
Source: Front Nutr. 2025 Sep 25;12:1623418. doi: 10.3389/fnut.2025.1623418 (PMC12507599; doi:10.3389/fnut.2025.1623418)
Supplement: Supplementary file 2 [file Data_Sheet_1.PDF]

Supplementary Material

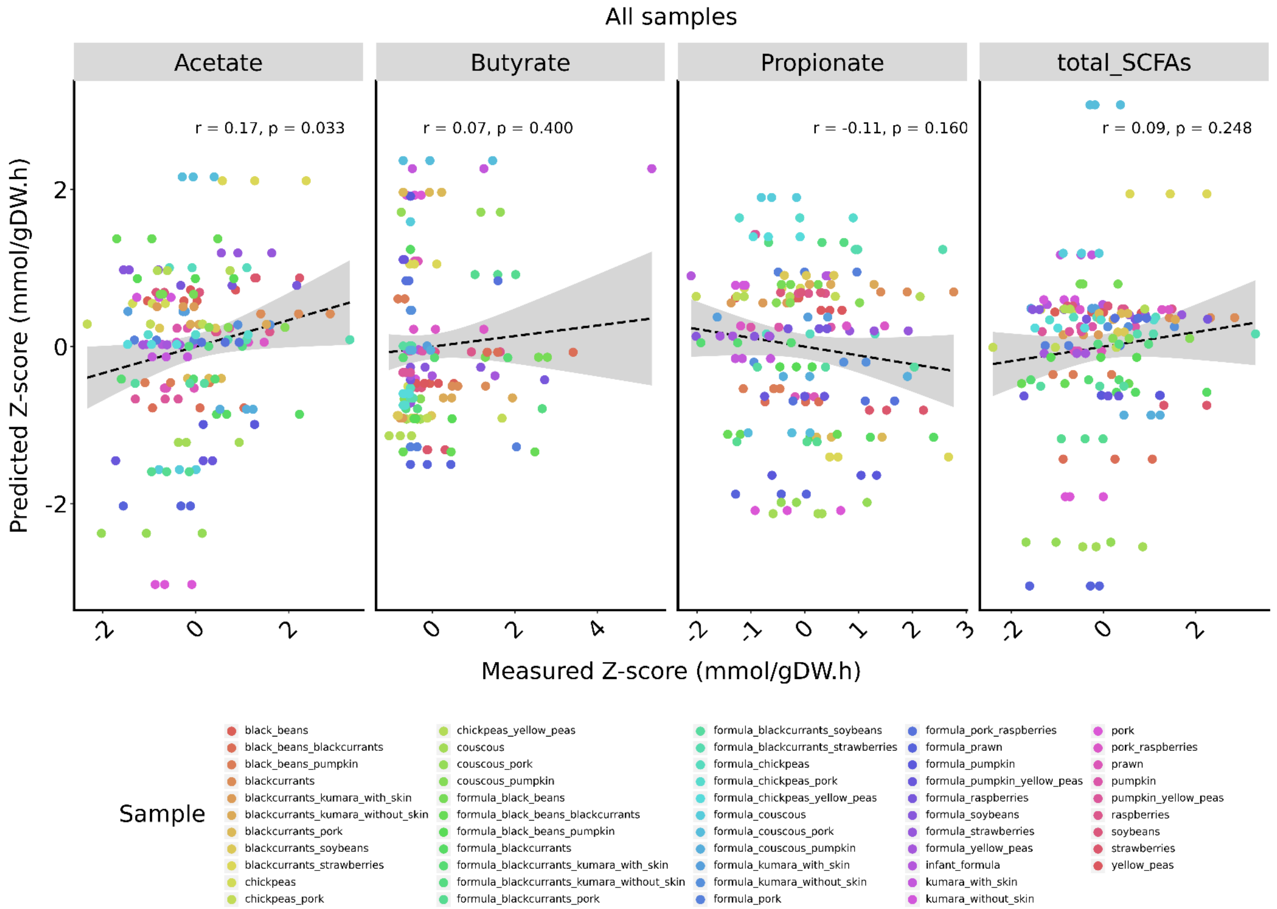

Supplementary Figure 1. Pearson correlations between measured and predicted z-scores. SCFAs are displayed from left to right as follows: acetate, butyrate, propionate. Total SCFAs correspond to the sum of acetate, propionate, and butyrate. Pearson correlation coefficients (r) and two-tailed p-values are calculated for each plot individually. A regression line is shown in black, with the corresponding 95% confidence interval in grey.

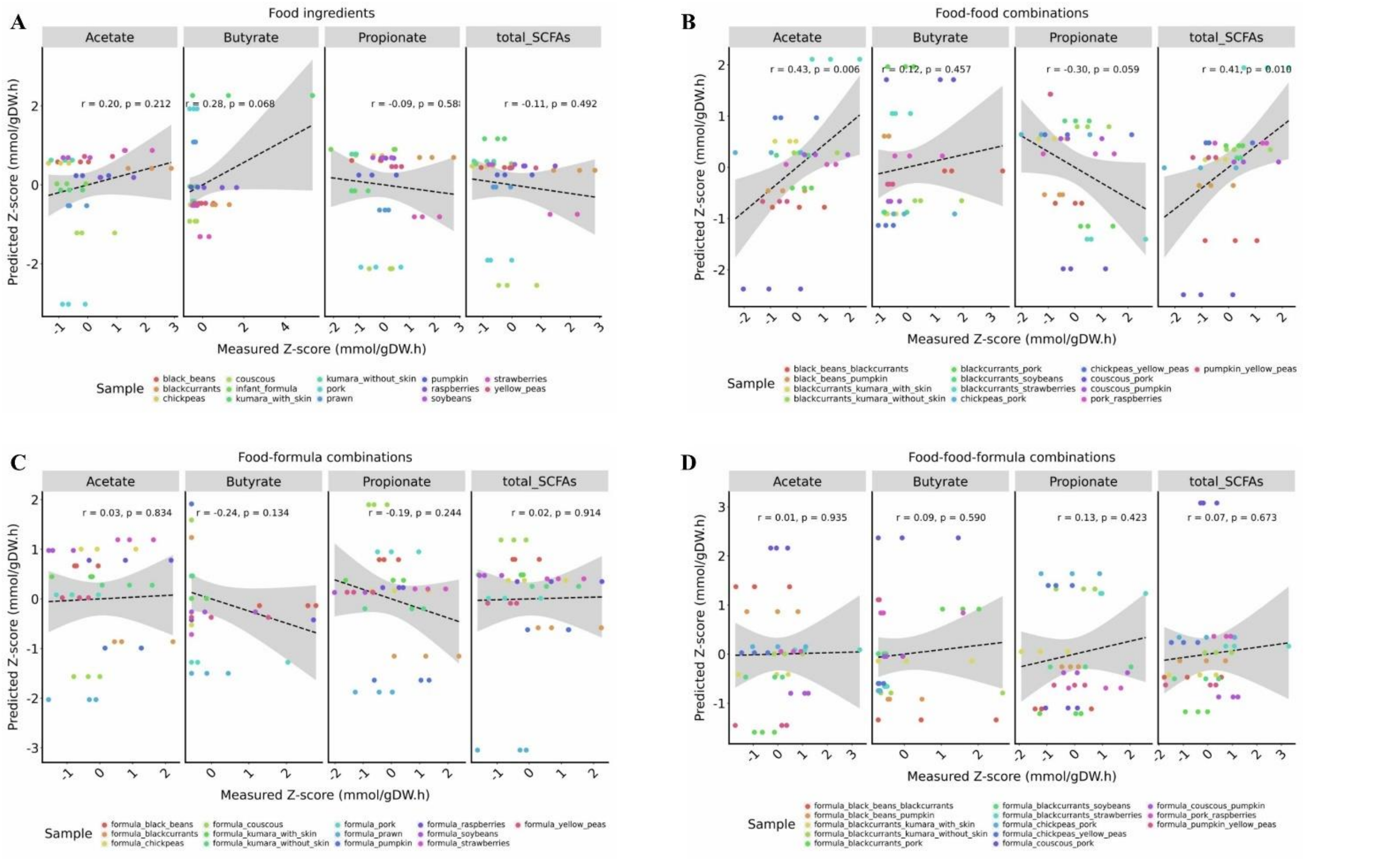

Supplementary Figure 2. Pearson correlations between measured and predicted z-scores of major SCFAs. Food ingredients (A), food-food combinations (B), food-formula combinations (C), and food-food-formula combinations (D). SCFAs are displayed from left to right as follows: acetate, butyrate, propionate. Total SCFAs correspond to the sum of acetate, propionate, and butyrate. Pearson correlation coefficients (r) and two-tailed p-values are calculated for each plot individually. A regression line is shown in black, with the corresponding 95% confidence interval in grey.

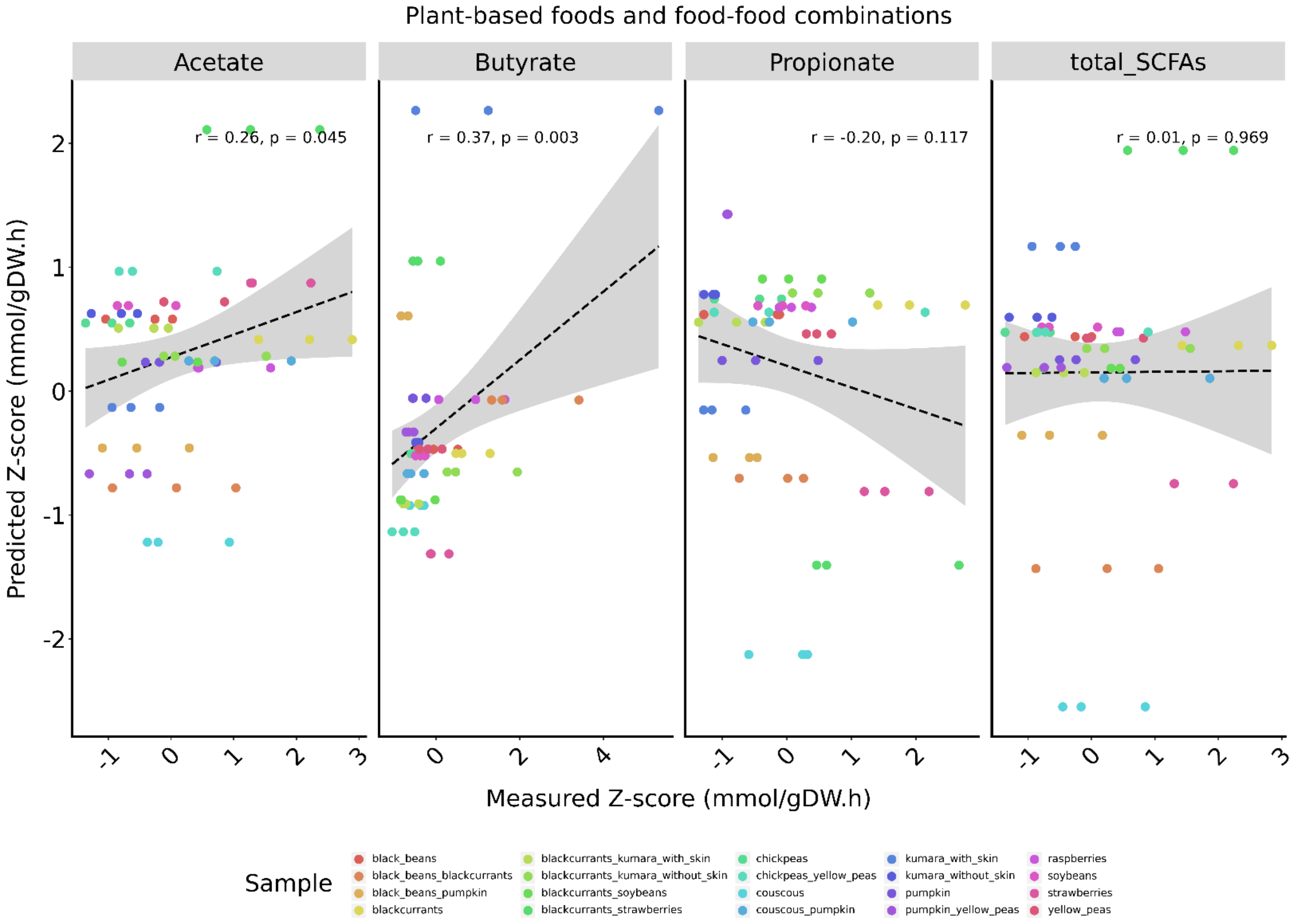

Supplementary Figure 3. Pearson correlations between measured and predicted z-scores of major SCFAs for plant-based food samples. SCFAs are displayed from left to right as follows: acetate, butyrate, propionate. Total SCFAs correspond to the sum of acetate, propionate, and butyrate. Pearson correlation coefficients (r) and two-tailed p-values are calculated for each plot individually. A regression line is shown in black, with the corresponding 95% confidence interval in grey.

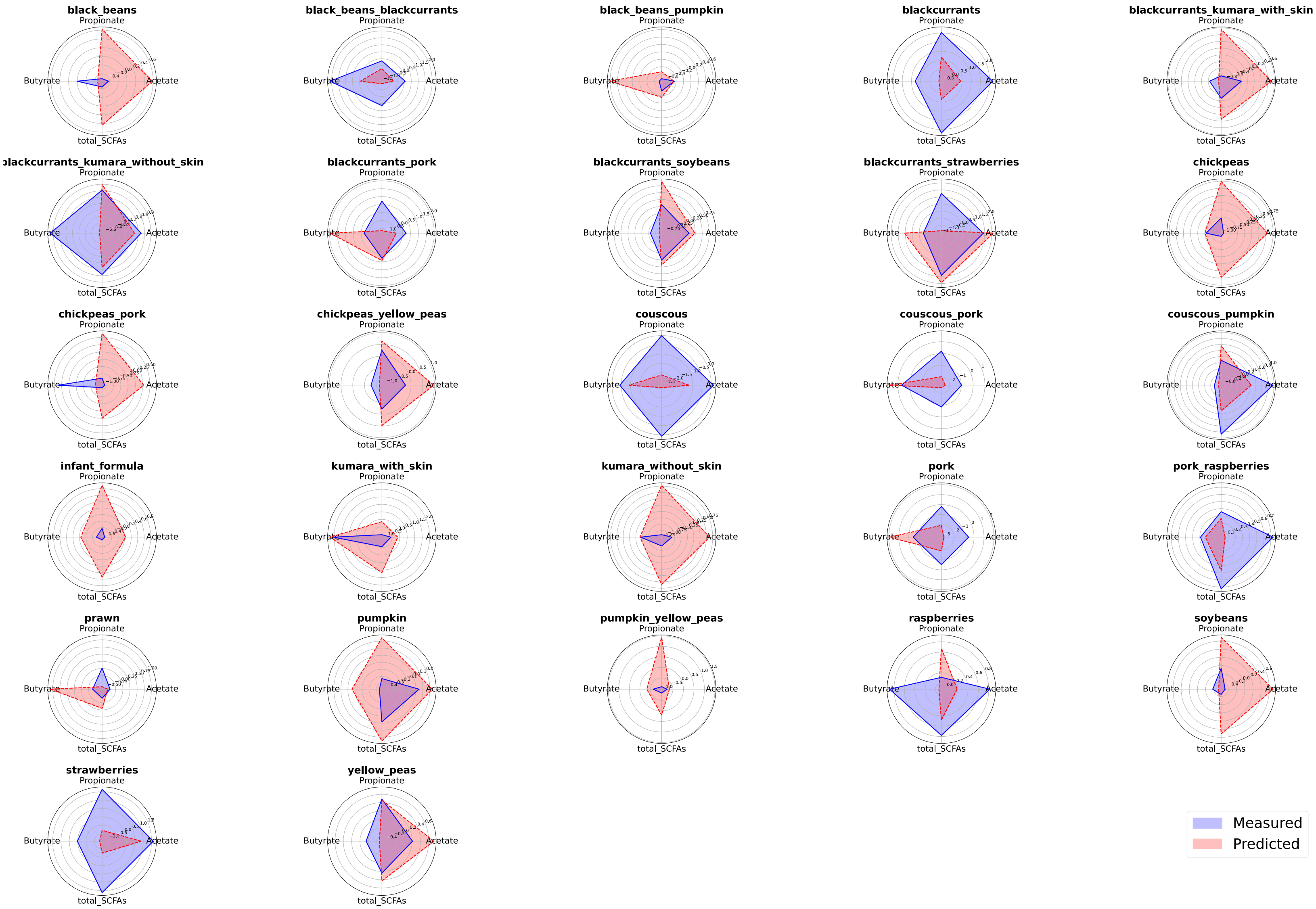

Supplementary Figure 4. Radar charts comparing measured and predicted z-scores for major SCFAs in food ingredients and food-food combinations. Total SCFAs correspond to the sum of acetate, propionate, and butyrate. Predicted values obtained via *in silico* modeling are displayed in red, while measured z-scores obtained via *in vitro* fermentation are shown in blue.

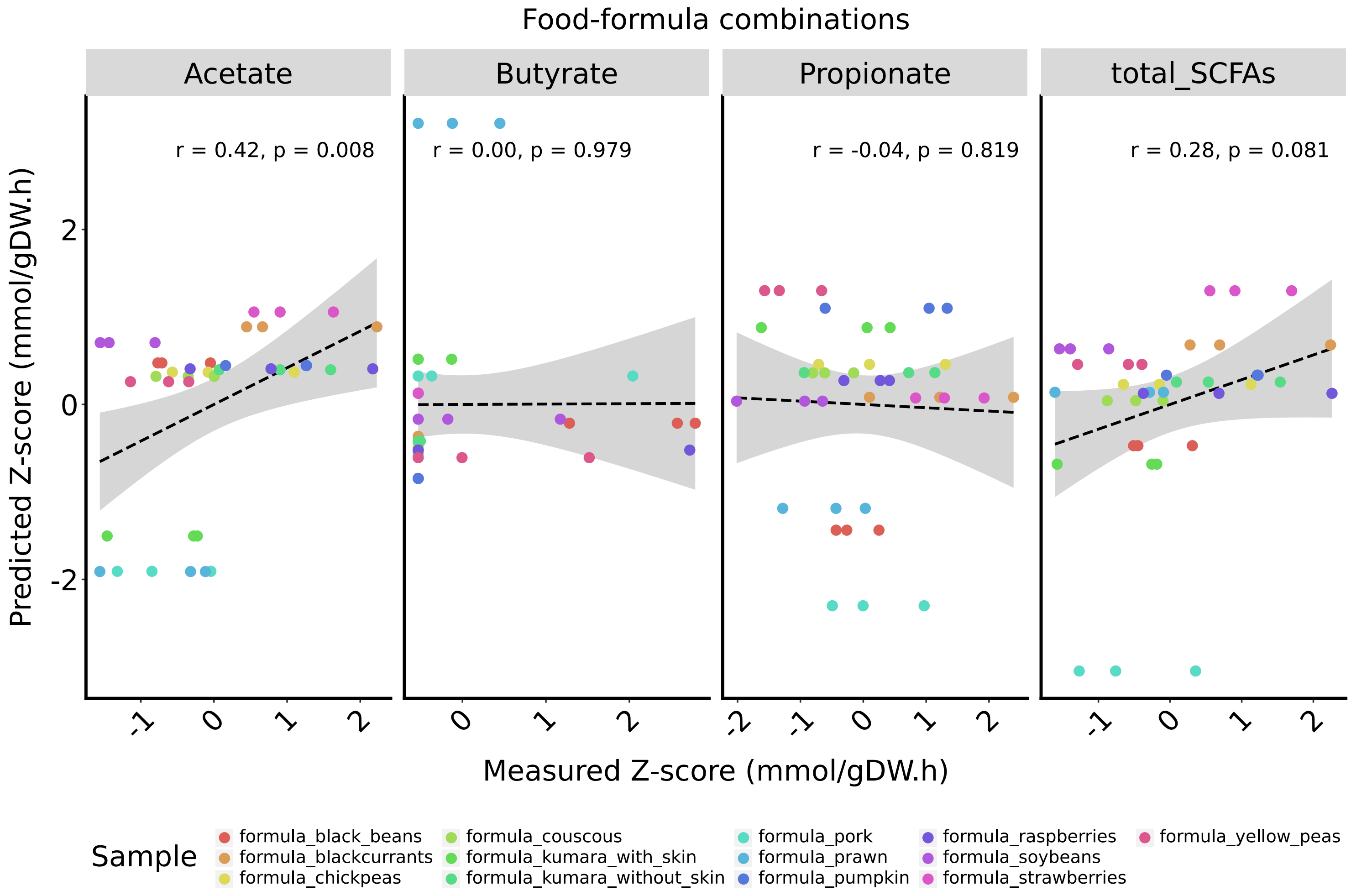

Supplementary Figure 5. Pearson correlations between measured and predicted z-scores of major SCFAs for food-formula samples. Simulations used post-fermentation microbial relative abundances for each sample. SCFAs are displayed from left to right as follows: acetate, butyrate, propionate. Total SCFAs correspond to the sum of acetate, propionate, and butyrate. Pearson correlation coefficients (r) and two-tailed p-values are calculated for each plot individually. A regression line is shown in black, with the corresponding 95% confidence interval in grey.

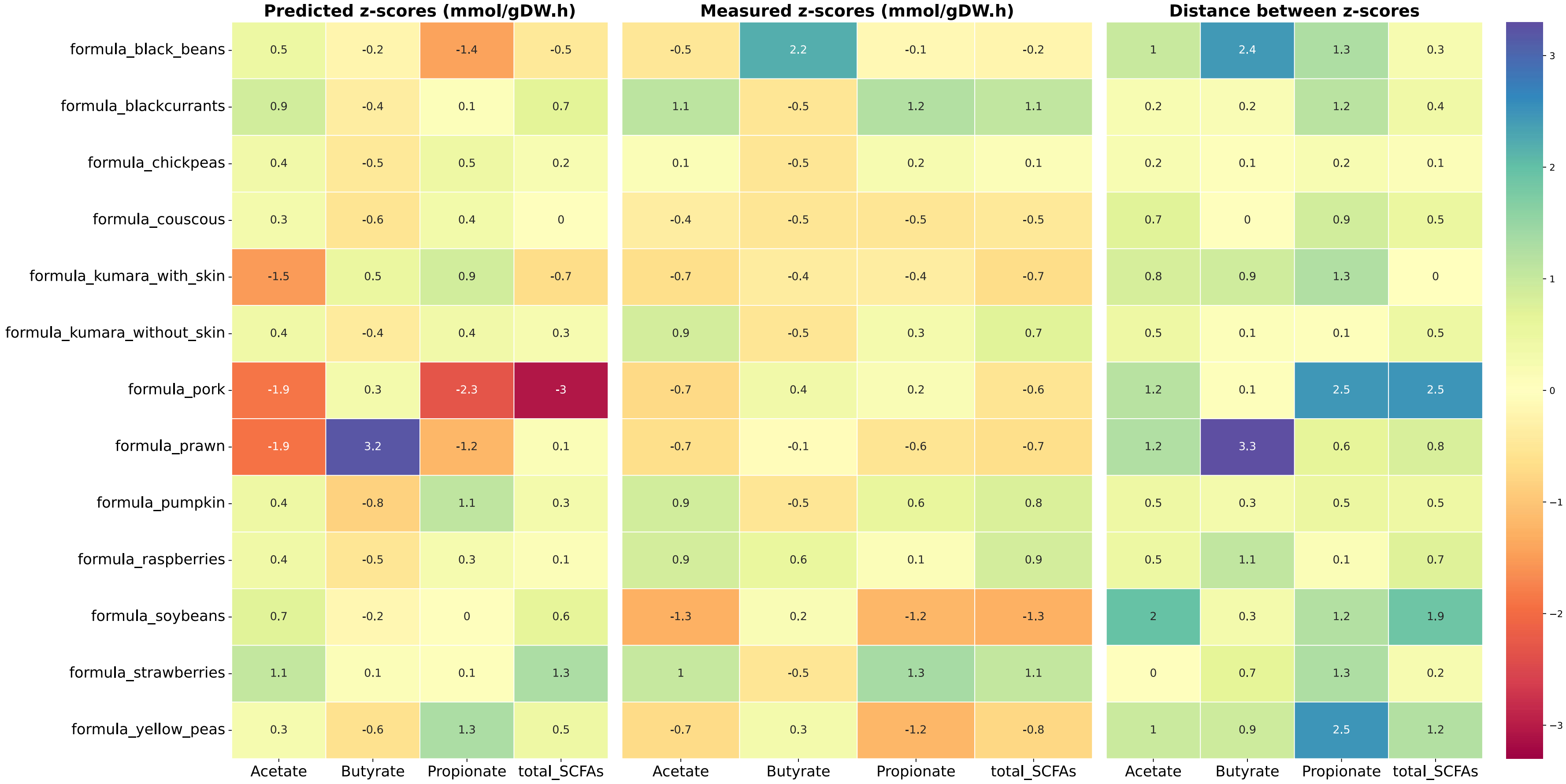

Supplementary Figure 6. Heatmap of measured and predicted z-scores of major SCFAs for food-formula combinations. Simulations used post-fermentation microbial relative abundances for each sample. Predicted z-scores obtained via *in silico* modeling are displayed on the left and measured z-scores obtained via *in vitro* fermentation on the middle. The absolute difference between predicted and measured z-scores is displayed on the right (distance between z-scores). Cells are coloured by intensity, with the lowest values in red and the highest values in blue.

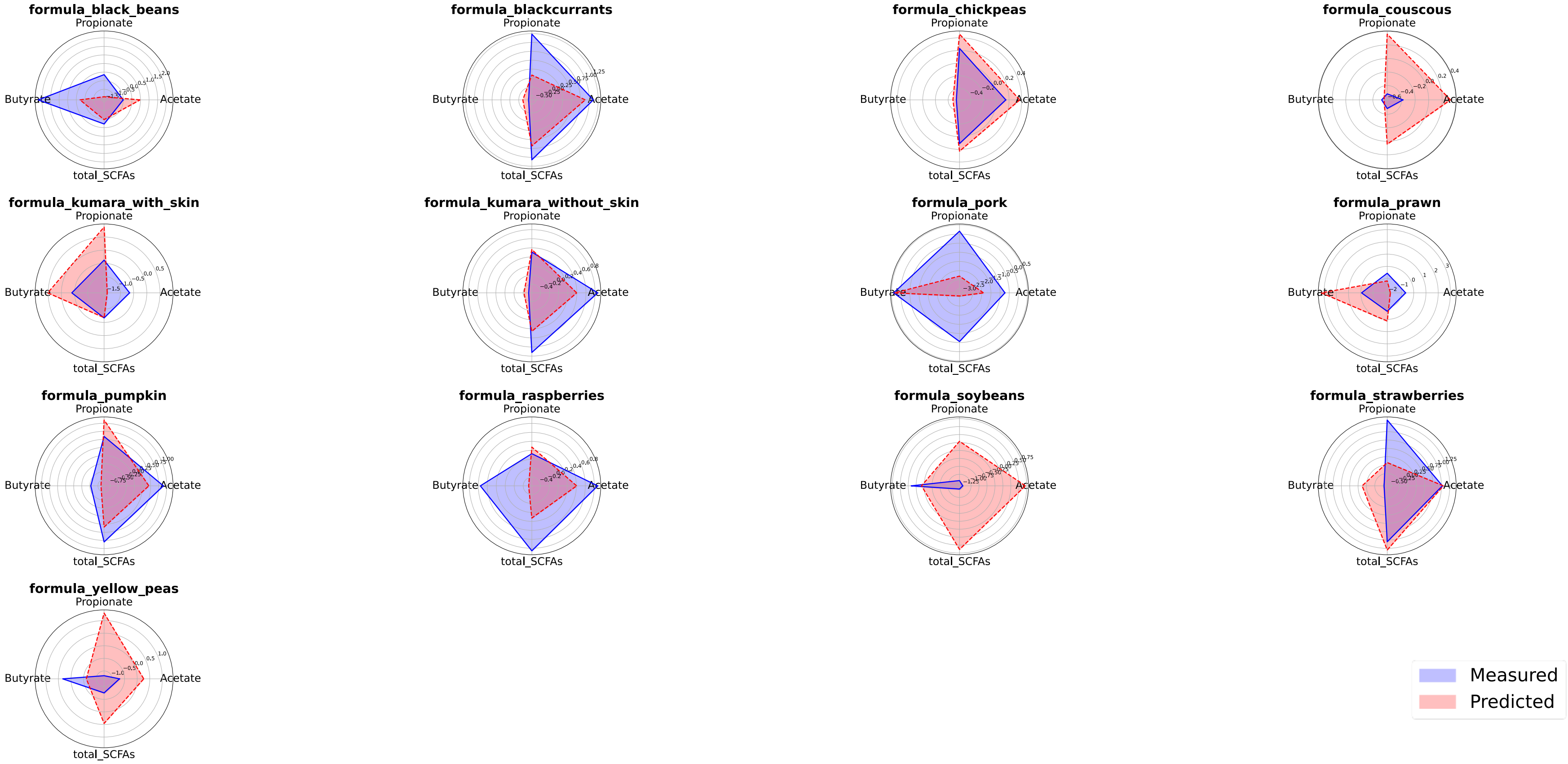

Supplementary Figure 7. Radar charts comparing measured and predicted z-scores for major SCFAs in food-formula combinations. Simulations used post-fermentation microbial relative abundances for each sample. Total SCFAs correspond to the sum of acetate, propionate, and butyrate. Predicted values obtained via *in silico* modeling are displayed in red, while measured z-scores obtained via *in vitro* fermentation are shown in blue.
